# Two Neuroanatomical Subtypes in Fibromyalgia Patients: Distinct Morphological Patterns and Treatment Outcomes

**DOI:** 10.1111/cns.70500

**Published:** 2025-07-01

**Authors:** Shiya Wu, Bin Jing, Yidan Wang, Meijuan Long, Yanting Li, Zihao Li, Juan Jiao

**Affiliations:** ^1^ Rheumatology Department Guang'anmen Hospital, China Academy of Chinese Medical Sciences Beijing China; ^2^ School of Biomedical Engineering Capital Medical University Beijing China

**Keywords:** fibromyalgia, gray matter volume, heterogeneity, sMRI, therapeutic response

## Abstract

**Objectives:**

To better investigate neurobiological heterogeneity in fibromyalgia for its symptom diversity and individual differences.

**Methods:**

We collected structural MRI data and clinical characteristics of Chinese female fibromyalgia patients and healthy controls matched by age and educational level, then invited qualified patients to undergo either Ba‐Duan‐Jin or pregabalin intervention for 12 weeks randomly. Structural MRI was analyzed by CAT12 software, and the regional volume of gray matter (GMV) was calculated according to the Brainnetome atlas. Fibromyalgia patients were clustered using the HYDRA algorithm to detect disease subtypes.

**Results:**

Two distinct neuroanatomical subtypes were found among 75 patients. Compared to 93 healthy controls, patients in subtype 1 (*n* = 38, 50.7%) showed widespread GMV increase, especially in some pain‐related brain regions, while no structural changes were observed in subtype 2 (*n* = 37, 49.3%). At the baseline before treatment, patients in subtype 1 showed a younger age (*p* = 0.037), longer illness duration (*p* = 0.042), and a severer psychological stress state evaluated by the Perceived Stress Scale (*p* = 0.008). After standardized treatment, subtype 1 patients showed less improvement in pain VAS score (*p* = 0.027) than subtype 2 patients. In addition, GMV of the bilateral dorsal caudate had negative correlations with stress level (Left *r* = −0.335, *p* = 0.040; Right *r* = −0.341, *p* = 0.036), and GMV of the left rostral temporal thalamus (*r* = 0.781, *p* = 0.038) and lateral amygdala (*r* = 0.761, *p* = 0.047) were positively related to the improvement of pain severity after treatment in subtype 1 patients.

**Conclusions:**

These two neuroanatomical subtypes in fibromyalgia emphasize different underlying neuropathological processes and need future studies to optimize individualized treatment.

**Trial Registration:**

ClinicalTrials.gov identifier: NCT03890133

## Graphical abstract

This study reveals two distinct neuroanatomical subtypes of fibromyalgia. Differences in clinical symptoms and treatment responses between two subtypes validate the subtype classification and highlight variations in the underlying neuropathological processes of fibromyalgia, offering insights for individualized treatment strategies.

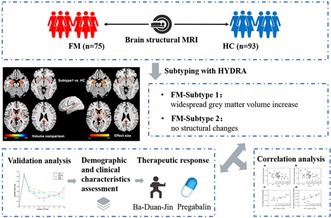

**Abbreviations**
ARI: Adjusted Rand Index
BDI‐II: Beck Depression Inventory‐II
FIQR: Revised Fibromyalgia Impact Questionnaire
GMV: volume of gray matter
HAMA: Hamilton Rating Scale for Anxiety
PSQI: Pittsburgh Sleep Quality Index
PSS: Perceived Stress Scale
sMRI: structural Magnetic Resonance Imaging
VAS: Visual Analogue Score
WPI: Widespread Pain Index

## 1 Introduction

Fibromyalgia is a worldwide chronic pain condition and its prevalence ranges from 0.7% to 9.3% [1]. In spite of the well‐known variability in clinical presentation and treatment response, fibromyalgia is a complex clinical syndrome mainly characterized by widespread pain, fatigue, non‐restorative sleep, stress, and emotional problems [2]. It has long been appreciated that such heterogeneity undermines the precision of clinical diagnosis and treatment guidelines. Still, research attempting to dissect such heterogeneity using the severity of fibromyalgia impact [3, 4], the key symptom severity combined with common complications [5], different emotional stimulus response [6], degree of symptomatic variation [7], and even patients' social support [8] subtyping had limited impact on clinical practice.

In the past decades, the rapid development of structural magnetic resonance imaging (sMRI) has played a crucial role in exploring the possible pathogenesis of fibromyalgia. However, the reported findings are quite different: some studies failed to find any gray matter alterations [9, 10], while other research found whole‐brain gray matter atrophies [11, 12] or gray matter enlargement in only specific brain regions [13, 14]. The latest meta‐analysis study found the GMV of the right postcentral gyrus, left angular gyrus, the right paracingulate gyrus and left rectus [15] were consistently aberrant in fibromyalgia. Although these seemingly inconsistent GMV alteration patterns in available sMRI studies may be partly due to the relatively small sample size (ranging from 10 to 32) [15], it actually underlines the necessity of investigating the neuroanatomical heterogeneity of fibromyalgia.

To our knowledge, sMRI has been recently performed to explore the heterogeneity of fibromyalgia based on clinical characteristics. One study revealed that individuals with moderate and moderate–severe symptoms exhibit the GMV increase in the right parahippocampal gyrus compared to patients with mild–moderate symptoms, while the GMV of the right middle cingulate cortex was inflated in patients with severe symptoms compared to patients with moderate–severe symptoms [3]. Another small‐sample study (*n* = 28) found that younger fibromyalgia patients (age < 50) showed increased GMV in the insula, putamen, and ventrolateral prefrontal cortex, while older patients (age = 50 or older) exhibited decreased gray matter in the anterior cingulate cortex, posterior cingulate cortex, and ventrolateral prefrontal cortex [16]. However, the current stratification of fibromyalgia patients is mainly based on subjective and extrinsic features rather than on objective and intrinsic characteristics such as brain neuroanatomical information.

In the present study, we collected a fibromyalgia cohort and used the brain morphological characteristics to detect the neuroanatomic heterogeneity by a data‐driven method. After that, comprehensive comparisons between the recognized two subtypes of fibromyalgia were conducted in both the pre‐treatment evaluations (demographic characteristics, disease severity indicators) and treatment outcomes. We hypothesize the distinct neuroanatomical subtypes of fibromyalgia may show some specific brain alterations, which could be used to explain the clinical diversity in fibromyalgia to some degree.

## 2 Methods

### 2.1 Study Sample and Image Acquisition

Female fibromyalgia patients and female healthy controls matched by age and educational level were collected from the Rheumatology department and Physical Examination Center of Guang'anmen Hospital from March 2019 to December 2021. The patients were confirmed to have fibromyalgia according to the 1990 and 2016 American College of Rheumatology criteria for the classification of fibromyalgia [17, 18], and had no less than 6 months of fibromyalgia duration to observe the long‐term structural alterations in the brain. Additionally, patients were required to not receive any treatments for fibromyalgia at least 4 weeks before the MRI examination to reduce therapeutic effects. The healthy controls were required to have no chronic pain. Exclusion criteria for fibromyalgia and healthy participants included: (1) left‐handedness assessed by the Edinburgh Handedness Inventory [19]; (2) severe depression or anxiety defined as either having a score of 16 or above on the Beck II Depression Inventory (BDI‐II) [20] or a score of 30 or above on the Hamilton Rating Scale for Anxiety (HAMA) [21]; (3) history of brain tumor, head injury, or cerebral vascular accidents; and (4) any other MRI contraindications.

The T1 images were acquired by a Siemens 3.0 T Skyra scanner (Siemens; Munich, Germany) using fast spoiled gradient‐echo sequence with the following parameters: TR = 5000 ms, TE = 2.98 ms, flip angle = 4°, FOV = 256 mm × 256 mm, slice thickness = 1 mm, no gap. The scanning for each patient lasted for 8 min. All participants signed an informed consent form. This study was approved by the Ethics Committee of Guang'anmen Hospital (approval number: 2019‐079‐ky) and was registered with ClinicalTrials.gov (registration number: NCT03890133).

### 2.2 Clinical Evaluation and Treatment

All fibromyalgia patients completed the case book, which includes two sections. Section 1 questioned patients about their demographic information and medical history. Section 2 consisted of a set of questionnaires assessing fibromyalgia including the pain severity and range assessed by Pain Visual Analogue Score (pain VAS) and Widespread Pain Index (WPI) [22], respectively, and the fatigue, sleep quality, depression, and perceived stress level evaluated by the Multidimensional Assessment of Fatigue Inventory (MFI‐20) [23], the Pittsburgh Sleep Quality Index (PSQI) [24], the Beck Depression Inventory‐II (BDI‐II) [25], and the Perceived Stress Scale (PSS) [26], respectively. The fibromyalgia impact was assessed by the revised Fibromyalgia Impact Questionnaire (FIQR) [27]. Higher scores reflect the greater severity of these clinical measures.

To investigate whether heterogeneity in brain structure underlies differential treatment responses among fibromyalgia patients, those 75 patients who had not practiced Ba‐Duan‐Jin, Tai Chi, yoga, or other forms of fitness exercise within the previous 12 months were invited to undergo either Ba‐Duan‐Jin exercise, a standardized non‐pharmacological therapy recommended by the latest Chinese fibromyalgia guideline [28], or pregabalin, one of the most frequently used drugs in fibromyalgia treatment, for 12 weeks randomly. Participants in the Ba‐Duan‐Jin group received guided Ba‐Duan‐Jin exercise plus a pregabalin placebo capsule, and those in the pregabalin group received pregabalin plus a program based on wellness education and muscle relaxation exercises. Both interventions were previously reported in detail [29]. The questionnaires in Section 2 of the case book were filled out again after completing the 12‐week treatment.

### 2.3 Image Preprocessing

The structural MRI images were processed with CAT 12 software (Version12.7, r1700). In detail, the voxel‐based morphometry pipeline includes initial and refined voxel‐based processing. The first part conducts SANLM denoising, resampling (into an isotropic voxel), bias correction, affine registration, and unified segmentation (into gray matter, white matter and cerebrospinal fluid) steps. The refined part contains segmentation refinement, skull stripping, regional parcellation, local intensity correction, AMAP/PVE segmentation, and Geodesic Shooting registration procedures. Finally, a normalized gray matter image with a 2.0 mm isotropic voxel size was generated for every subject and then smoothed by a 4 mm FWHM Gaussian kernel. Of note, the patients with low image quality rating were discarded for following analysis.

### 2.4 Subtyping Fibromyalgia With HYDRA

Subsequently, the whole brain was parcellated into 246 regions according to the Brainnetome Atlas (https://atlas.brainnetome.org/), and the volume of each region was calculated for each subject. The HYDRA algorithm [30] was utilized on the ROI volume information to identify fibromyalgia subtypes with age and total intracranial volume as covariates. HYDRA can determine the cluster number (*K* = 2–10) hyperplanes to generate convex polyhedra for separating fibromyalgia patients from the healthy controls, and every patient will be assigned to the hyperplane closest to themselves and eventually divided into *K* clusters. The clustering stability was quantified by the adjusted rand index (ARI) in a 5‐fold cross‐validation manner. Finally, the cluster number (*K* = 2) corresponding to the highest ARI was used to differentiate the fibromyalgia patients.

### 2.5 Validation Analysis of Clustering Results

The cluster analysis was validated by selecting another brain atlas AAL3, and HYDRA was again used to access and compare the cluster results of fibromyalgia patients with the original results. In addition, the cluster result was also verified by a half‐split test [31], that is, the whole dataset (including fibromyalgia patients and healthy controls) was randomly divided into two halves (split1 dataset: 37 fibromyalgia patients and 46 healthy controls; and split2 dataset: 38 fibromyalgia patients and 47 healthy controls), and HYDRA was applied on the sampled data to verify the consistency with the original cluster results.

### 2.6 Voxel‐Wise Volume Alterations in Fibromyalgia Subtypes

The divided subtypes of fibromyalgia patients were respectively compared with the healthy controls in whole brain gray matter volume. The statistical threshold for group difference was set at FDR corrected *p* < 0.05 and cluster size > 40 voxels. For comparison purposes with ungrouped fibromyalgia patients, the whole brain gray matter volume of total fibromyalgia patients was also compared with those of the healthy controls using the same statistical threshold.

### 2.7 Clinical Examination of Fibromyalgia Subtypes

Data were presented as mean (SD) for continuous variables and frequency (%) of participants for categorical variables. Two‐sample t‐tests were used to assess differences between subtypes in age, illness duration, education years, the fibromyalgia key symptoms severity (the scores of WPI, Pain VAS, PSS, BDI‐II, PSQI, and MFI‐20), and the impact of fibromyalgia (FIQR total score) at baseline and post‐treatment. Results are presented as between‐group differences with 95% confidence intervals. Within each subtype, the relationships of gray matter volumes of altered brain regions with PSS score at baseline were assessed by partial correlation, and the relationships between gray matter volumes of altered brain regions and the change of pain VAS after treatment were also assessed using partial correlation analysis after adjusting for age, symptom duration, and baseline pain VAS and PSS scores.

## 3 Results

**FIGURE 1:**
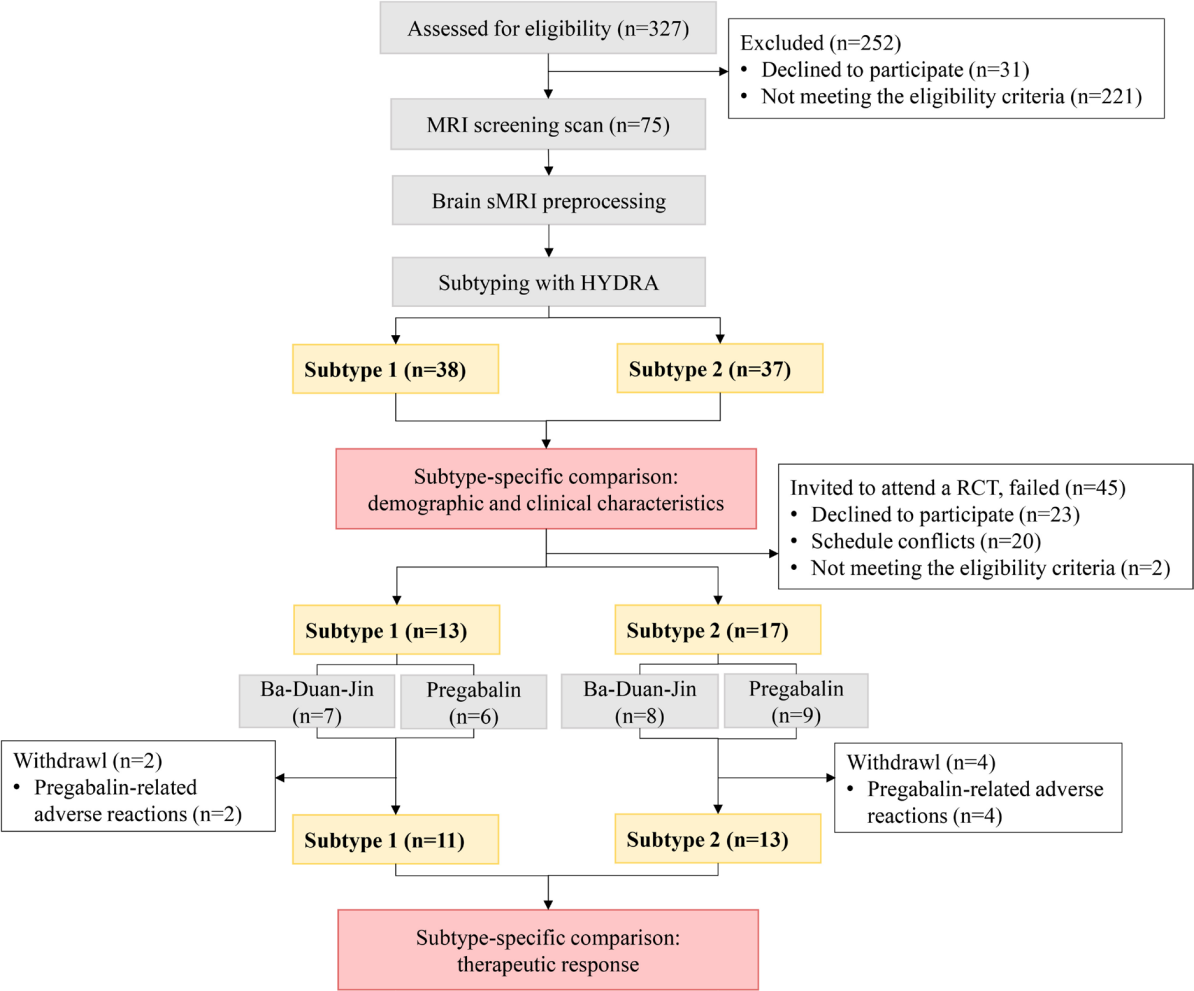
Flow diagram of the study.

A total of 75 female fibromyalgia patients and 93 female healthy controls met eligible criteria and completed MRI screening. Among these 75 patients, 73 patients qualified for intervention and were invited, but only 30 patients consented successfully, and at last, 24 patients completed the 12‐week intervention (see Figure 1).

### 3.1 HYDRA Reliably Reveals Two Neuroanatomical Subtypes in Fibromyalgia

**FIGURE 2:**
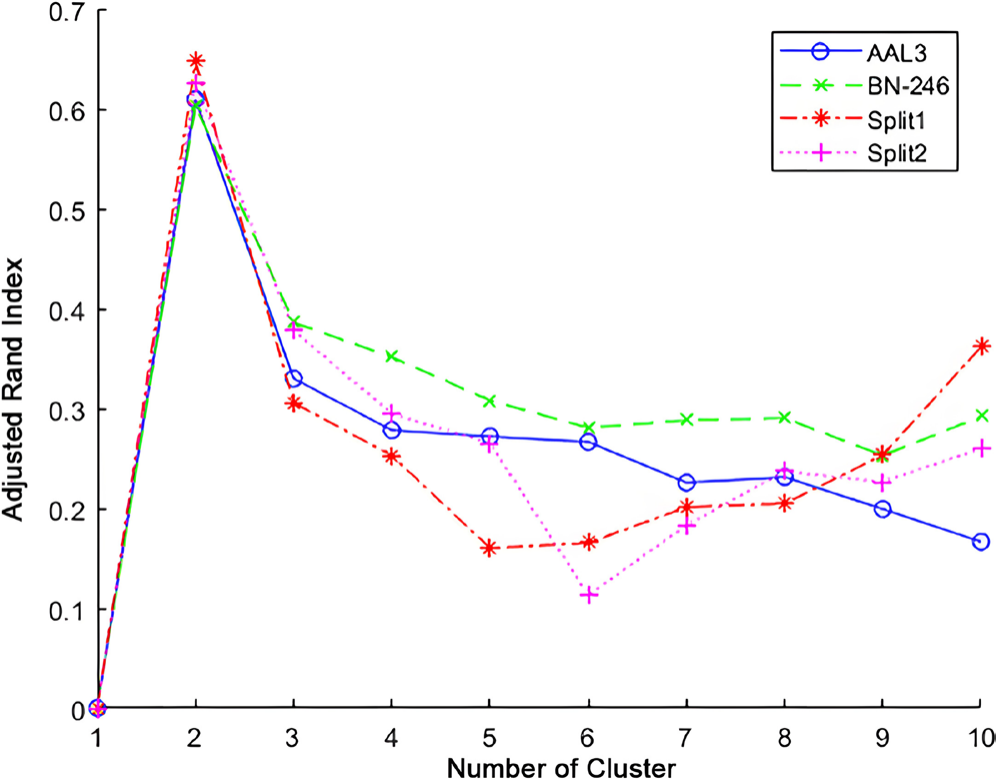
The adjusted rand index (ARI) value in HYDRA.

When applying HYDRA on the brain volume from the Brainnetome atlas, *K* = 2 was found to show the highest ARI (see Figure 2) in comparison to other cluster results. Moreover, the validation analysis revealed that the cluster number of *K* = 2 consistently showed the highest ARI by using AAL3 atlas or using half‐split verification. When using AAL3 atlas, 72 (98.6%) fibromyalgia subjects were reported with the same cluster results as BN‐246 atlas, while only 1 (1.4%) fibromyalgia subjects were assigned into conflicting subtypes. In addition, 2 (5.4%) fibromyalgia subjects in split1 dataset and 2 fibromyalgia (5.3%) patients in split 2 dataset were determined with the inconsistent clustering results in comparison to the original BN‐246 results.

### 3.2 The Morphological Deficits in Two Subtypes of Fibromyalgia Are Quite Different

**FIGURE 3:**
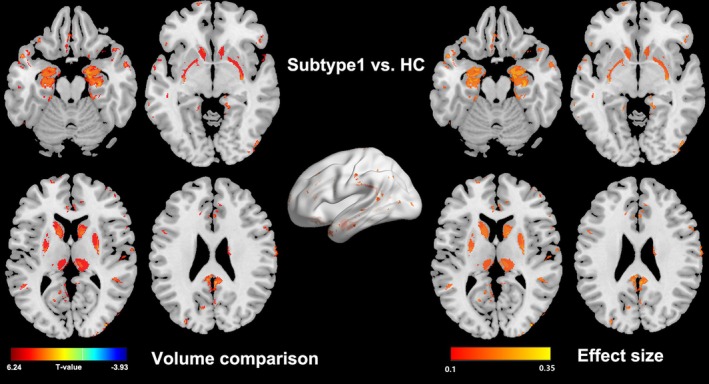
Gray matter volume in subtype 1 compared to healthy controls (HC). The color bar in the left panel stands for the *T* values, and the color bar in the right panel means the Cohen's *d* value.

Subtype 1 showed a widespread GMV increase compared to healthy controls (see Figure 3), and the abnormalities were mainly located at pain related regions such as the caudate, putamen, thalamus, hippocampus, amygdala, precuneus, and parahippocampus. In contrast, subtype 2 didn't show any morphological alteration in comparison to healthy controls. Interestingly, when comparing the GMV between the whole fibromyalgia patients and the whole healthy controls, there are also not any significant alterations.

### 3.3 The Divergent Demographic and Disease Characteristics Exist in Two Subtypes of Fibromyalgia

As presented in Table 1, subtype 1 patients had a younger age (*p* = 0.037), a longer disease duration (*p* = 0.042), and a severe level of psychological pressure (*p* = 0.008) than subtype 2 patients. No significant difference was found in educational level, pain range and severity, depression, insomnia, fatigue, and somatic function.

**TABLE 1:** Demographic and clinical characteristics comparison between subtype 1 and 2.^a^

Characteristics	Subtype 1 (*n* = 38)	Subtype 2 (*n* = 37)	*p*
Age (years)	43.3 (10.5)	48.4 (10.1)	0.037
Symptom duration (month)	79.7 (69.5)	51.9 (48.9)	0.042
Years of education	14.3 (3.1)	13.5 (3.1)	0.27
Pain VAS	6.1 (1.3)	6.2 (1.8)	0.73
WPI	11.1 (3.9)	11.3 (3.6)	0.78
MFI‐20	69.5 (12.9)	65.6 (16.0)	0.25
PSQI	11.9 (2.9)	11.0 (3.7)	0.25
BDI‐II	9.6 (6.8)	7.9 (7.1)	0.15
PSS	30.7 (9.0)	25.0 (9.3)	0.008
FIQR	43.4 (17.2)	35.2 (20.1)	0.06

Abbreviations: BDI‐II, beck depression inventory‐II; FIQR, revised fibromyalgia impact questionnaire; MFI‐20, multidimensional fatigue inventory‐20; Pain VAS, Pain Visual Analogue Scale; PSQI, Pittsburgh Sleep Quality Index; PSS, Perceived Stress Scale; WPI, widespread pain index.

^a^ All values are means (±SD).

### 3.4 The Post‐Treatment Outcomes of the Two Subtype Groups Are Distinct

There were no differences in baseline demographic and clinical characteristics between the two subtypes among the 24 patients who completed 12‐week intervention (Table S1), and no differences were found between Ba‐Duan‐Jin and pregabalin groups in the change of pain VAS or in other treatment outcomes (Table S2). As presented in Table 2, patients in subtype 1 exhibited less improvement in pain VAS than patients in subtype 2 (*p* = 0.027). No difference was found between the two subtypes in other treatment outcomes including WPI, MFI‐20, PSQI, BDI‐II, PSS, and FIQR.

**TABLE 2:** Treatment outcome changes from baseline in Subtype 1 and 2.^a^

Outcome changes	Subtype 1 (*n* = 11)	Subtype 2 (*n* = 13)	*p*
Pain VAS	−2.6 (−3.9 to −1.2)	−4.1 (−4.8 to −3.4)	0.027
WPI	−2.4 (−6.3 to 1.6)	−5.9 (−9.6 to −2.1)	0.17
MFI‐20	−24.3 (−32.4 to −16.1)	−22.6 (−29.0 to −16.2)	0.73
PSQI	−2.4 (−5.25 to 0.5)	−3.1 (−5.7 to −0.4)	0.69
BDI‐II	−3.6 (−6.9 to −0.4)	−4.6 (−6.9 to −2.4)	0.58
PSS	−10.9 (−17.2 to −4.6)	−7.4 (−13.2 to −1.6)	0.37
FIQR	−9.2 (−20.0 to 1.5)	−18.9 (−32.4 to −5.5)	0.24

Abbreviations: BDI‐II, Beck Depression Inventory‐II; FIQR, Revised Fibromyalgia Impact Questionnaire; MFI‐20, Multidimensional Fatigue Inventory‐20; Pain VAS, Pain Visual Analogue Scale; PSQI, Pittsburgh Sleep Quality Index; PSS, Perceived Stress Scale; WPI, Widespread Pain Index.

^a^ All values are means (95% confidence interval).

### 3.5 The GMV of Specific Regions Are Related to PSS Score and the Change of Pain VAS

**FIGURE 4:**
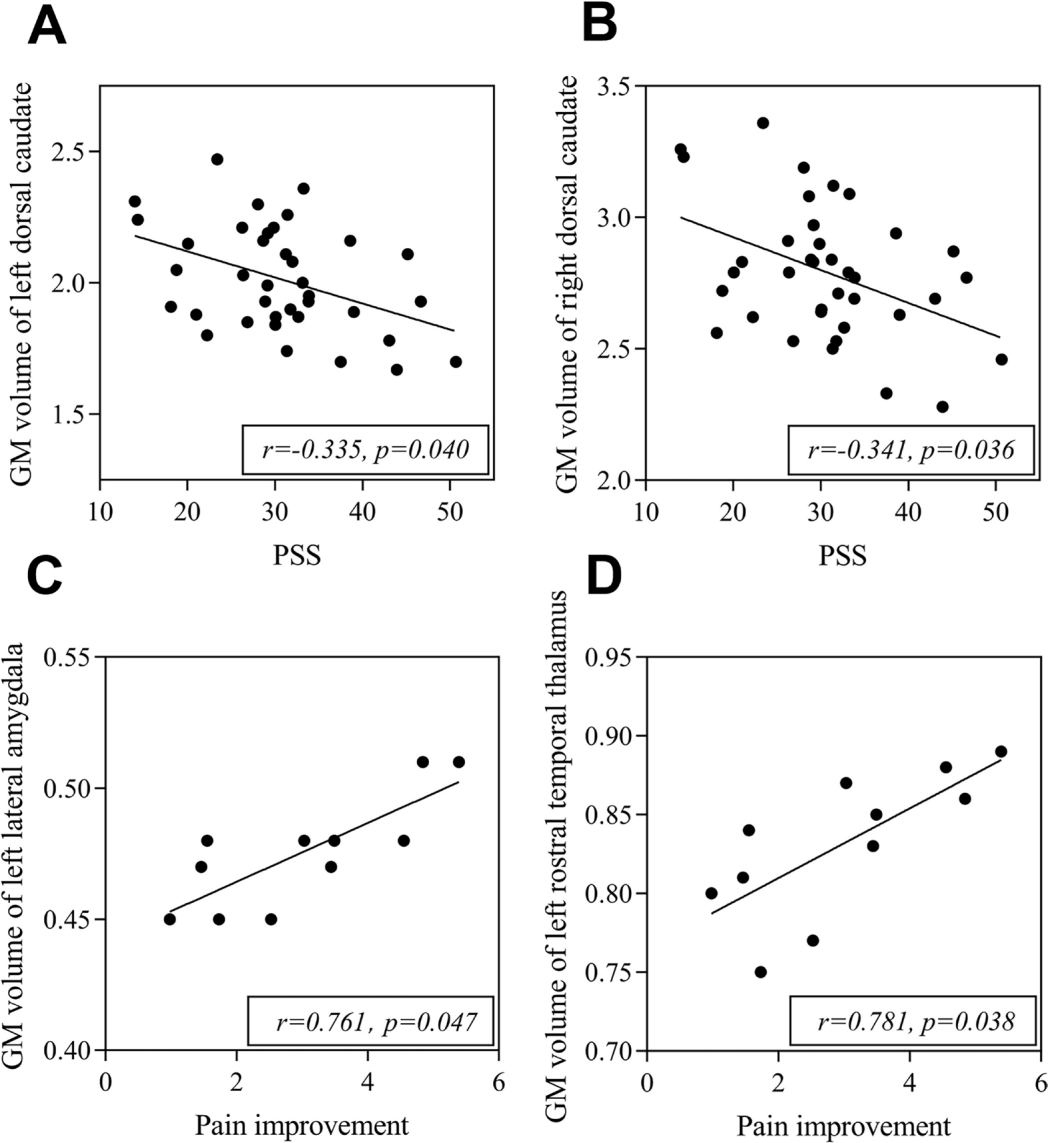
Correlations between the Perceived Stress Scale (PSS) and gray matter (GM) volume of bilateral dorsal caudate (A: Left, B: Right) in subtype 1, correlations between pain improvement assessed by the change of pain VAS post‐treatment and GMV of (C) left rostral temporal thalamus, and of left lateral amygdala (D) in subtype 1 patients.

After adjusting for age, symptom duration, and baseline pain VAS score, GMV of bilateral dorsal caudate had a negative relationship to PSS (Left: *r* = −0.335, *p* = 0.040; Right: *r* = −0.341, *p* = 0.036) (Figure 4), and left rostral temporal thalamus (*r* = 0.781, *p* = 0.038) and left lateral amygdala (*r* = 0.761, *p* = 0.047) were positively correlated with the change of pain VAS after adjusting for the above factors and baseline PSS score in subtype 1 patients (Figure 4). However, no significant correlation between GMV of those specific brain regions and clinical evaluation was found in subtype 2 patients. Moreover, no significant correlation was found between symptom duration and GMV in altered brain regions in both subtype 1 and 2 patients.

## 4 Discussion

This study revealed two neuroanatomical subtypes of fibromyalgia using brain structural imaging, and the two subtypes showed different demographic and clinical characteristics, brain alteration patterns, and treatment outcomes for the first time. Moreover, the study revealed the deficit of standard case–control comparisons that the average brain structural differences of total patients obscured the presence of neuroanatomical subtypes. Our findings suggest fibromyalgia displays clear neuroanatomical heterogeneity, indicating the distinct underlying neurobiological mechanisms, which sheds light on the personalized optimization of therapeutic outcomes.

The most important finding in current study was that fibromyalgia patients in subtype 1 showed widespread GMV increase, particularly in the caudate, putamen, thalamus, hippocampus, amygdala, precuneus, and parahippocampus, which are key regions related to pain, and patients in subtype 2 didn't display any significant GMV in comparison to healthy controls. The GMV findings in subtype 1 patients are opposite to some studies that reported individuals diagnosed with fibromyalgia only exhibited widespread gray matter atrophy [11, 12], and are partially consistent with other studies that claimed the fibromyalgia patients showed both GMV reduction and increase in specific‐regions [13, 14]. The latest meta‐analysis also reported the increase of GMV in several brain regions [15], although the detailed regions were not fully consistent with our findings in this study. At the same time, our study in subtype 2 patients clarifies the recent reports of no change in GMV [9, 10]. Moreover, accumulating evidence imply that the long‐term unremitting symptoms of pain may induce disruptions mainly in the limbic system [32], which has also been validated in the study.

Using the intrinsic brain morphometric characteristics, subtype 1 patients manifested higher psychological stress, longer illness duration, and younger age in comparison to subtype 2 patients. Though fibromyalgia is commonly considered a stress‐related disorder characterized by chronic non‐inflammatory widespread pain, there was seldom direct evidence of the relationship between brain GMV and stress evaluation in fibromyalgia patients. Evidence has shown that both chronic occupational stress and stressful life events can induce volumetric alterations in specific subcortical regions, including the amygdala and caudate nucleus [33, 34], and the variations of brain may return to a normal state at follow‐up [34]. An animal model also demonstrated the causal effect of stress exposure on fibromyalgia‐like pain development [35]. The finding of the negative relationship between stress perception and the volume of the dorsal caudate in the female fibromyalgia population in the current study, together with previous findings of a negative association between pain tolerance and the dorsal caudate in chronic pain patients [36], demonstrates the core role of the dorsal caudate in the stress‐motivational aspects of chronic pain disorder.

Compared with patients in subtype 2, only patients in subtype 1 displayed the associations between the therapeutic performance and the GMV of the left rostral temporal thalamus and lateral amygdala. They had positive impacts on the analgesic effect of body–mind exercise and pharmacotherapy. The rostral temporal thalamus covers parts of the anterior thalamic complex, which projects to limbic areas in the medial temporal lobe and parts of the dorsomedial thalamic nucleus relating to emotional behavior, memory, attention, and higher cognitive functioning of organization and planning [37]. The lateral amygdala is regarded as the sensory input gateway, receiving information from both the thalamus and cortical areas, and is a sensory input channel that receives multiple sensory signals, including auditory, visual, and somatosensory stimuli, especially the key brain region that processes associative fear memories [38].

The perception of pain is associated with the activity of multiple brain regions—nociceptive stimuli activating core structures of “pain network” including the thalamus and the amygdala, which are all highly related to pain processing. Structural changes have been observed in the thalamus and amygdala in different pain conditions varied from smaller [39, 40], larger [11, 41], to no change [9, 10] in patients with chronic pain. Furthermore, the volume of the thalamus and amygdala alters disparately post‐treatment in patients with chronic pain. A study reported a 4‐week interdisciplinary pain management program resulting in increased volumes in the thalamus and amygdala with clearly improved symptoms [42]. On the contrary, patients who experienced trigeminal neuralgia recurrence after initial pain relief showed right amygdala enlargement compared to patients with long‐lasting pain relief [43]. Despite the paradoxical link between structural changes and pain reduction, it is certain that brain structure has the potential to predict treatment performance, and that differences in analgesic effect between the two subtypes may be related to different neuropathological mechanisms. According to the existing literature [36, 44], we speculate that decreased activity of the mesolimbic dopaminergic circuit might be the possible underlying neuropathological processes that lead subtype 1 patients to be less responsive to current intervention.

Limitations of this study include: (1) the enrolled patients in the study were all single‐center female fibromyalgia with disease course of more than 6 months, and it is unknown whether these subtypes are suitable for multi‐center, male patients or female patients with less than 6 months duration; (2) structural MRI was not retested after the 12‐week intervention to explore the reversibility of volume changes in those brain regions, though some clinical interventions reversed the brain structural in fibromyalgia [45]; (3) Due to scheduling conflicts and refusal to participate mainly, the number of patients successfully accepted Ba‐Duan‐Jin and pregabalin intervention is small. High quality designed clinical intervention trail is still needed to verify the current finding that GMV alterations associating with pain improvement.

In conclusion, our results provide new insights into the underlying biological mechanisms of the heterogeneity in fibromyalgia, which might be helpful to deepen the understanding of neuropathological alterations in fibromyalgia. The current findings demonstrated that the two distinct neuroanatomical subtypes have relationships with some clinical profiles and to some extent the treatment effects. It can be corroborated that the individuals with fibromyalgia have distinct underlying neurobiological mechanisms, which may guide the optimization of therapeutic program.

## Author Contributions

Conception and design: J.J. Acquisition of data: Y.W., M.L., Y.L., Z.L, S.W. Analysis and interpretation of data: S.W., B.J., Y.W., M.L., J.J. Drafting of the manuscript: S.W., B.J., Y.W., J.J. Final approval of the article: all authors.

## Ethics Statement

The authors confirm that any aspect of the work covered in this manuscript that has involved fibromyalgia patients has been conducted with the ethical approval approved by the ethics committee of Guang'anmen Hospital and that it follows the guidelines of the Declaration of Helsinki for humans (approval number:2019‐079‐ky). No AI‐related tools were used in the writing of the article.

## Conflicts of Interest

The authors declare no conflicts of interest.

## Supporting information


Data S1.

## Data Availability

The data that support the findings of this study are available from the corresponding author upon reasonable request.
