# Supplementary material for: Two Neuroanatomical Subtypes in Fibromyalgia Patients: Distinct Morphological Patterns and Treatment Outcomes
Source: CNS Neurosci Ther. 2025 Jul 1;31(7):e70500. doi: 10.1111/cns.70500 (PMC12209714; doi:10.1111/cns.70500)
Supplement: Supplementary file 1 — Data S1. [file CNS-31-e70500-s001.docx]

**Supplementary Table 1. Demographic and baseline clinical characteristics comparison between Subtype 1 and Subtype 2 patients who completed 12-week intervention***

| Characteristics | Subtype 1 (n=11) | Subtype 2 (n=13) | P value |
| --- | --- | --- | --- |
| Age (years) | 45.1 (11.7) | 49.9 (10.8) | 0.31 |
| Symptom duration (month) | 83.4 (68.1) | 74.0 (67.3) | 0.69 |
| Years of education | 14.9 (2.4) | 12.4 (3.2) | 0.09 |
| Pain VAS | 5.5 (1.6) | 6.2 (1.8) | 0.29 |
| WPI | 10.1 (5.0) | 11.3 (2.9) | 0.49 |
| MFI-20 | 73.8 (7.6) | 67.7 (9.6) | 0.10 |
| PSQI | 11.3 (3.0) | 10.8 (3.4) | 0.71 |
| BDI-Ⅱ | 10.7 (5.8) | 9.4 (8.6) | 0.66 |
| PSS | 34.4 (7.9) | 27.8 (8.5) | 0.06 |
| FIQR | 37.0 (12.9) | 38.6 (19.9) | 0.82 |

*All values are means (±SD). Abbreviation: Pain VAS: Pain Visual Analogue scale; WPI: widespread pain index; MFI-20: Multidimensional Fatigue Inventory-20; PSQI: Pittsburgh Sleep Quality Index; BDI-Ⅱ: Beck Depression Inventory-II; PSS: Perceived Stress Scale; FIQR: revised Fibromyalgia Impact Questionnaire.

Supplementary Table 2. Treatment outcome changes from baseline in Ba-Duan-Jin group and pregabalin group*

| Outcome changes | Ba-Duan-Jin group (n=15) | Pregabalin group (n=9) | P value |
| --- | --- | --- | --- |
| Pain VAS | -3.2 (-4.4 to -2.1) | -3.6 (-4.3 to -3.0) | 0.61 |
| WPI | -5.3 (-8.5 to -2.0) | -2.6 (-7.6 to 2.5) | 0.32 |
| MFI-20 | -23.5 (-29.1 to -18.0) | -23.1 (-33.5 to -12.8) | 0.93 |
| PSQI | -2.6 (-5.0 to -0.2) | -3.0 (-6.4 to 0.4) | 0.83 |
| BDI-Ⅱ | -3.6 (-6.0 to -1.2) | -5.1 (-8.3 to -2.0) | 0.41 |
| PSS | -10.2 (-15.7 to -4.8) | -7.0 (-13.9 to -0.2) | 0.42 |
| FIQR | -11.2 (-18.1 to -4.2) | -20.0 (-41.8 to 1.8) | 0.30 |

*All values are means (95% confidence interval). Abbreviation: Pain VAS: Pain Visual Analogue scale; WPI: widespread pain index; MFI-20: Multidimensional Fatigue Inventory-20; PSQI: Pittsburgh Sleep Quality Index; BDI-Ⅱ: Beck Depression Inventory-II; PSS: Perceived Stress Scale; FIQR: revised Fibromyalgia Impact Questionnaire.
